# Let’s Agree to Disagree on Operative Versus Nonoperative Treatment for Distal Radius Fractures in Older People: Protocol for a Prospective International Multicenter Cohort Study

**DOI:** 10.2196/52917

**Published:** 2024-02-13

**Authors:** Nicole Maria van Veelen, Bryan J M van de Wall, Ruben J Hoepelman, Frank F A IJpma, Björn-Christian Link, Reto Babst, Rolf H H Groenwold, Detlef van der Velde, Nadine Diwersi, Mark van Heijl, R Marijn Houwert, Frank J P Beeres

**Affiliations:** 1 Department of Orthopaedic and Trauma Surgery Luzerner Kantonsspital Luzern Switzerland; 2 Department of Health Sciences and Medicine University of Lucerne Luzern Switzerland; 3 Department of Trauma Surgery Universitair Medisch Centrum Utrecht Utrecht Netherlands; 4 Department of Trauma Surgery Universitair Medisch Centrum Groningen Groningen Netherlands; 5 Department of Clinical Epidemiology Universitair Medisch Centrum Leiden Leiden Netherlands; 6 Department of Trauma Surgery St. Antonius Ziekenhuis Utrecht Netherlands; 7 Department of General and Trauma Surgery Kantonsspital Obwalden Sarnen Switzerland; 8 Department of Surgery Diakonessenhuis Utrecht Utrecht Netherlands

**Keywords:** distal radius fracture, older patients, natural experiment, study protocol, observational study

## Abstract

**Background:**

Distal radius fractures are the most frequently encountered fractures in Western societies, typically affecting patients aged 50 years and older. Although this is a common injury, the best treatment for these fractures in older patients is still under debate.

**Objective:**

This prospective study aims to compare the outcome of operatively and nonoperatively treated distal radius fractures in the older population. Only patients with distal radius fractures for which equipoise regarding the optimal treatment exists will be included.

**Methods:**

This prospective international multicenter observational cohort study will be designed as a natural experiment. Natural experiments are observational studies in which treatment allocation is determined by factors outside the control of the investigators but also (largely) independent of patient characteristics. Patients aged 65 years and older with an acute distal radius fracture will be considered for inclusion. Treatment allocation (operative vs nonoperative) will be based on the local preferences of the treating hospital either in Switzerland or the Netherlands. Hence, the process governing treatment allocation resembles that of randomization. Patients will be identified after treatment has been initiated. Based on the radiographs and baseline information of the patient, an expert panel of 6 certified trauma surgeons from 2 regions will provide their treatment recommendation. Only patients for whom the experts disagree on treatment recommendations will ultimately be included in the study (ie, for whom there is a clinical equipoise). For these patients, both operative and nonoperative treatment of distal radius fractures are viable, and treatment choice is predominantly determined by personal or local preference. The primary outcome will be the Patient-Rated Wrist Evaluation score at 12 weeks. Secondary outcomes will include the Physical Activity Score for the Elderly, the EQ questionnaire, pain, the living situation, range of motion, complications, and radiological outcomes. By including outcomes such as living situation and the Physical Activity Score for the Elderly, which are not relevant for younger cohorts, valuable information to tailor treatment to the needs of the older population can be gained. According to the sample size collection, which was based on the minimal important clinical difference of the Patient-Rated Wrist Evaluation, 92 patients will have to be included, with at least 46 patients in each treatment group.

**Results:**

Enrollment began in July 2023 and is expected to continue until summer 2024. The final follow-up will be 2 years after the last patient is included.

**Conclusions:**

Although many trials on this topic have previously been published, there remains an ongoing debate regarding the optimal treatment for distal radius fractures in older patients. This observational study, which will use a fairly new methodological study design, will provide further information on treatment outcomes for older patients with distal radius fractures for which to date equipoise exists regarding the optimal treatment.

**International Registered Report Identifier (IRRID):**

DERR1-10.2196/52917

## Introduction

Distal radius fractures are the most common fractures encountered in Western societies, and they typically occur in patients aged 50 years and older [1,2]. Despite being so common, there remains an ongoing discussion on the best treatment for these fractures, especially in older patients, ranging from nonoperative treatment to complex surgery [3].

Previous studies have used varying definitions of “older people” and based this only on chronological age. However, the spectrum of physical demands and capabilities is wide among patients of the same age, which will likely be reflected in the satisfaction and outcome after treatment of distal radius fractures. Additionally, even frail patients may rely on a good function of the wrist to maintain independence if they, for example, require walking aids, and therefore need to fully weight bear on the upper extremity.

This study aims to compare the outcome of operatively and nonoperatively treated distal radius fractures in the older population by evaluating functional and radiological outcomes as well as range of movement, quality of life, and change in independence or living situation. By evaluating the quality of life and patient independency or living situation, we aim to gain more insight into outcomes after distal radius fracture in older patients while considering their individual functional demands.

Therefore, we have designed this study as a prospective international multicenter cohort study in which only patients with distal radius fractures for which equipoise regarding the optimal treatment exists will be included. The primary objective will be to compare clinical outcomes between older patients with distal radius fracture treated operatively and those treated nonoperatively.

## Methods

### Study Design

Although randomized controlled trials are generally considered the gold standard to investigate the effects of medical treatments, both patients and surgeons can have a strong preference for a certain treatment, which may limit the feasibility of a randomized controlled trial and therefore the generalizability of its results [4,5]. Provided possible incomparability of patients who receive different treatment modalities (ie, confounding) is adequately controlled for, observational studies could provide an alternative source of information.

A natural experiment design uses existing variation in treatment allocation (eg, due to practice variation). Natural experiments are observational studies in which treatment allocation is determined by factors outside the control of the investigators but also (to a large extent) independent of patient characteristics [6,7]. We assume the decisions regarding the treatment of distal radius fractures are largely influenced by the training of the treating surgeons. Since patients with a distal radius fracture generally visit the nearest hospital, treatment allocation is dependent on which surgeon is on call, which is likely independent on patient characteristics. Hence, the process governing treatment allocation arguably resembles that of randomization. Practice variation therefore provides an opportunity to conduct a natural experiment of the effect of operative versus nonoperative treatment of distal radius fractures. In order to further minimize the influence of patient characteristics on treatment decisions, a selection could be made of those patients for whom a panel of physicians disagree regarding the preferred treatment option (ie, for whom there clearly is a clinical equipoise). For these patients, both operative and nonoperative treatment of distal radius fractures are viable, and treatment choice is predominantly determined by personal or local preference.

This study is an international multicenter prospective observational cohort study for which patients will be identified after treatment has been initiated. Outcome data will be prospectively collected. Patients will be recruited from 5 hospitals in countries with a predominant preference for operative (Switzerland) and nonoperative treatment (the Netherlands).

### Patient Population

Local investigators will review the list of patients seen in the emergency departments of the participating hospitals to identify eligible patients. The inclusion criteria are all patients 65 years and older with an acute (<14 days after injury) distal radius fracture treated at one of the participating hospitals.

The exclusion criteria are no informed consent provided; patients transferred after initial operative treatment at a nonparticipating hospital; delayed presentation (>14 days after injury); insufficient follow-up (<12 months) or unavailable to follow-up due to residency in other hospital area; concomitant injury to the ipsilateral or contralateral upper extremity; cognitive impairment precluding answering questionnaires; non-German, non-English, and non-Dutch speaking; preexisting comorbidities that preclude operative treatment; pathological fractures; open fractures; and neurovascular injury requiring operative treatment.

### Ethical Considerations

This study was approved by the ethics committee of Northwest and Central Switzerland (ID 2022-00142). It will be conducted in accordance with the Declaration of Helsinki and the principles of Good Clinical Practice. The study has been registered on ClinicalTrials.gov (ID NCT05631314). The protocol was written in adherence to the SPIRIT (Standard Protocol Items: Recommendations for Interventional Trials) guideline. A signed informed consent form is required prior to patient inclusion. The patients will be informed about the aim, content, and requirements of the study by the study leader or delegate either during an initial presentation or at a routine outpatient clinic appointment and will receive an information and consent form. The patients will be given the opportunity to ask questions and clarify any issues prior to giving their consent. No compensation or payments will be provided to project participants. Project data will be handled with the utmost discretion. They will be coded and a file to decode these data will be saved in the research folder only accessible by the principal investigator and project leaders.

### Expert Panel

Following the identification of eligible patients and obtainment of informed consent, anonymous radiographs including key images of computer tomography scans if available, and baseline characteristics including information regarding comorbidities and activity levels (details below) will be made available on a secure web-based platform. This will allow the members of the panel to reach a “clinical” decision regarding their treatment recommendation. The panel will be blinded to the actual treatment received and the origin of the case.

The comorbidities, which will be reported to the experts, are cardiomyopathy including valvular disease, history of stroke or other brain injury with persistent neurological deficit, diabetes mellitus, cardiovascular disease, active malignancy, rheumatoid arthritis, and history of alcohol or drug abuse.

Additionally, the experts will be provided with information regarding the usage of the following medications: anticoagulant agents (vitamin K antagonists and new oral anticoagulants), immunosuppressants, and current chemotherapy.

The activity levels are independent, independent with support from family or district nurse or nursing home, and use of a walking aid prior to injury (no, walking stick, frame, and not mobile).

The expert panel will consist of 3 representatives from each “school.” All panel members will be certified trauma surgeons. Half of them are generally in favor of conservative treatment (“school A”), while the others are generally in favor of operative treatment (“school B”). Patients will ultimately be included in the study if clinical equipoise is achieved, meaning 2 or more of the experts disagree with the rest of the panel. This is expected to result in 2 comparable groups for which clinical equipoise exists. The actual treatment the patient receives will continue as initially planned by the treating physician (see below).

### Intervention

The decision whether operative or nonoperative treatment is chosen is left to the treating trauma surgeon as per the local standard of the participating hospital. Treatment will be initiated before the patient is approached for recruitment. Nonoperative treatment will consist of immobilization in a below the elbow cast for 4-6 weeks with or without prior closed reduction.

Operative intervention will be based on the treating surgeon’s experience and preference. It will consist of open reduction and internal plate fixation using volar, dorsal, spanning or a combination of plates, or reduction (open or closed) and fixation with an external fixator with or without Kirschner wires. The participating hospitals in Switzerland will use the 2.4-mm distal radius plate system (Arthrex) for internal plate fixation. Perioperative management including anesthesia, antibiotics, and thromboembolism prophylaxis will follow the national guidelines. Postoperative care and immobilization will be decided by the treating surgeon. When possible, functional aftercare without immobilization will be allowed. Physiotherapy will be provided according to the local standards.

All patients (operative and nonoperative) will be reviewed in the outpatient clinic at 6 and 12 weeks as well as 1 year after treatment or surgery. In addition, patients will be contacted by phone 2 years after treatment or surgery.

### Outcomes

#### Primary Outcome Measure

The primary outcome is the Patient-Rated Wrist Evaluation (PRWE) score measured at 12 weeks. This is a 15-item questionnaire that measures wrist pain and disability in activities of daily living. The score ranges from 0 to 100 with the best score being 0 [8,9].

#### Secondary Outcome Measures

##### Functional Outcome

Secondary functional outcome measures will include the PRWE at 1 year, Physical Activity Score for the Elderly, the Numerical Rating Scale for pain, and the EQ-5D-5L questionnaire. The Physical Activity Score for the Elderly is a questionnaire with multiple choice and open questions. The score combines information on household, leisure, and occupational activities. Based on duration, frequency, and intensity level of activities performed the previous week, a score is calculated ranging from 0 to 793, with higher scores reflecting more physical activity [10]. The EQ-5D-5L questionnaire is a questionnaire that measures general health status, with higher scores reflecting a better quality of life. It is based on 5 dimensions (mobility, self-care, usual activities, pain or discomfort, and anxiety or depression), which are rated on a scale with 5 levels. It also includes the EQ visual analogue scale for patients to state their self-rated health on a scale from 0 to 100, with 0 being the worst health imaginable [11]. The Numerical Rating Scale is a widely used scale to measure pain intensity, ranging from 0 (no pain) to 10 (worst pain imaginable) [4]. The living situation will be assessed at the first presentation, 6 weeks, 12 weeks, 1 year, and 2 years after injury. It will be defined as independent, independent with support from family, district nurse or similar, and nursing home. Range of motion of the wrist (dorsal extension, palmar-flexion, ulnar-, radial-abduction, pro-, and supination) as well as the finger-palm-distance will be assessed 12 weeks after injury. Additionally, the duration of surgery and the occurrence of intraoperative complications will be documented.

##### Complications

Complications will be assessed at every outpatient visit and after 2 years. They will include infection, nonunion, implant failure, complex regional pain syndrome, and any adverse event needing surgical intervention. For patients treated surgically, the need for implant removal will also be recorded. Infections will be defined according to the guidelines of The Centers for Disease Control and Prevention and subdivided into superficial incisional and deep infections. Deep infections will include both deep incisional and organ or space infections [5]. The diagnosis of complex regional pain syndrome is based on the Budapest clinical diagnostic criteria [12]. Nonunion is defined as a Radius union scoring system score greater than or equal to 6. It will be assessed 1 year after injury. The RUSS score evaluates union on dorso-volar and lateral radiographs by assigning a score of 0 to 2 on each cortex. Zero points are given if the fracture line is visible with no callus, 1 point if a callus is present but the fracture line is still visible, and 2 points if the fracture line is not visible and a bridging callus is present. In surgically treated cases, where the cortex is not visible due to the implant, a score of 0 is given to that cortex [13]. In case of doubt with regard to union, a computer tomography scan will be acquired. Patients with nonunion will be divided into 2 groups based on their symptoms (symptomatic vs asymptomatic).

##### Radiological Outcome

X-rays will be obtained as per standard hospital protocol at 6 and 12 weeks as well as 1 year after injury. The final radiological outcome will be determined 1 year after injury and will include union, radial inclination, volar tilt, carpal alignment, ulnar variance, and the presence and size of any step or gap in the articular surface.

### Timeline and Recruitment

**Figure 1:**
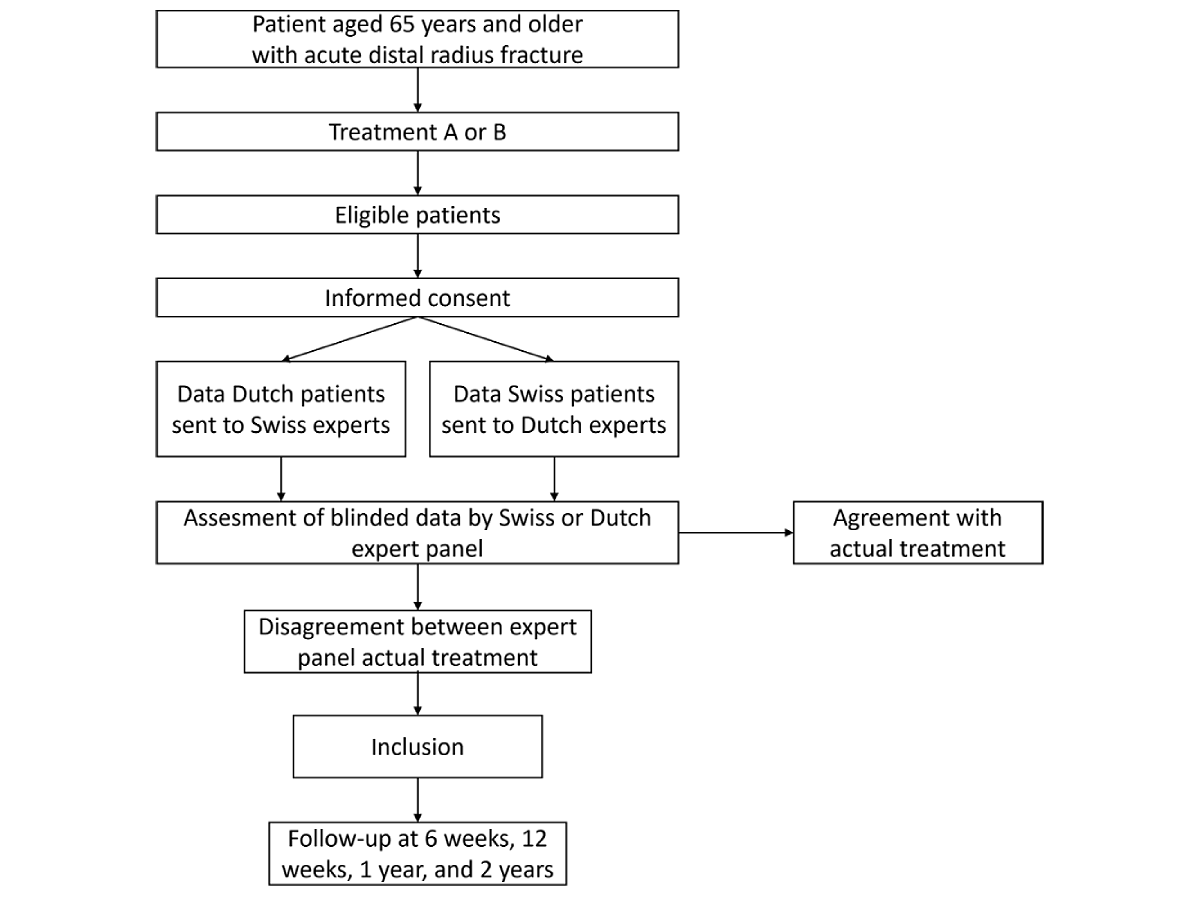
Flowchart of Patient Selection.

The recruitment procedure is visualized in Figure 1. The follow-up schedule for the patients and time point for the assessment of each outcome are listed in Table 1.

**Table 1:** Outcome measures.

	First presentation	6 weeks	12 weeks	1 year	2 years
Outpatient clinic		✓	✓	✓	
X-ray	✓	✓			
PRWE^a^	✓		✓	✓	✓
PASE^b^	✓		✓	✓	✓
NRS^c^ pain		✓	✓	✓	✓
ROM^d^			✓		
EQ-5D-5L	✓		✓	✓	✓
Complications		✓	✓	✓	✓
Living situation	✓	✓	✓	✓	

^a^PRWE: Patient-Rated Wrist Evaluation.

^b^PASE: Physical Activity Score for the Elderly.

^c^NRS: Numerical Rating Scale.

^d^ROM: range of movement.

### Data Collection

In every participating institution, a project leader will fill in all the patient information in the secure web-based database on REDCap (Research Electronic Data Capture; Vanderbilt University). The original data will be collected from the institution’s electronic patient records, the picture archiving and communication system, the different questionnaires, and pseudonymized prior to entry in the database.

Health care workers from the participating institutions will get access to REDCap, which enables centralized tracking of potentially eligible patients in compliance with the good clinical practice guidelines for electronic data collection. Every 2-4 weeks, this list of patients will be screened by the project leader, and explanatory and response variables will be entered on the study website. All data will be saved in a research folder that can only be accessed by the principal investigator and the project leader or leaders.

### Sample Size Calculation

This study will be a noninferiority study, for which we assume that conservative treatment in older patients with a distal radius fracture is noninferior to operative treatment. The reason for choosing a noninferiority design is as follows. Operative treatment for distal radius fractures inherently exposes patients to additional risks of operation-related complications and costs. This is not the case for conservative treatment. If the study would demonstrate that both treatments are equal with regard to the primary outcome, it would automatically imply that patients should be treated with the lesser invasive treatment of the 2 (in this case, conservative treatment).

The sample size is based on the minimal important clinical difference of the PRWE score, which is 11.5 points (with an average SD 14). Using the minimal important clinical difference (11.5 points) as the noninferiority margin, a statistical significance threshold (α) of 5%, and a power of 90%, 32 patients per group are needed [14]. Taking into account a 30% loss to follow-up (due to the considerable age of the included study population), a total of 92 patients will have to be included, with at least 46 patients in each treatment group.

### Statistical Analysis

The statistical software package SPSS (IBM Corp) will be used for analysis. All analyses will be performed according to the intention-to-treat principle. Multiple imputation will be used for missing values. Depending on their distribution, baseline characteristics will be described as means and SDs or median and IQR for continuous variables. Categorical variables will be reported as counts and percentages. Treatment groups will be compared using an independent Student *t* test (2-tailed) or chi-square test, as appropriate.

The primary outcome will be analyzed using a regression analysis with the 12 weeks PRWE score as dependent and treatment as independent variable. Potential confounders (notably age and fracture type) will be included as covariates in the model. Regression coefficients will be calculated with corresponding 95% CI. Regression analysis will also be used for analyzing the secondary outcomes with correction for the previously mentioned confounders.

## Results

Patient enrollment started both in the Netherlands and Switzerland in July 2023. To date, 23 patients have given their consent in Switzerland and 59 in the Netherlands. The results of the first expert panel decisions are currently pending. Depending on the rate of equipoise, enrollment is expected to be completed after approximately 1 year. The final follow-up will be 2 years after the inclusion of the last patient.

## Discussion

Despite many trials on this topic, the optimal treatment for distal radius fractures in older patients remains debated, and regional preferences continue to influence the treatment chosen. This provides the perfect situation to perform a natural experiment [6]. The Let’s Agree to Disagree on Operative Versus Nonoperative radius study is a prospective international multicenter cohort study that will use a fairly new methodological study design [6]. One of the main benefits of this study design is that patients will be treated as per the local standards. We expect this to lead to high participation rates and prevent the negative effects of a learning curve. By limiting inclusion to those patients, for whom the expert panel disagrees on the treatment method, only those cases in which equipoise is attained are evaluated. We anticipate the 2 patient groups to be comparable as natural randomization based on geographical location will occur. Therefore, confounding is expected to be minimal. Additionally, multivariable analysis will be performed.

Many studies on the treatment of distal radius fractures in older patients have been published and yet no sound conclusion can be drawn. While some authors demonstrated a benefit of volar plating, others were unable to show a difference in functional outcomes between operative and nonoperative treatment [15-19]. Previous studies examined functional outcome based on patient-reported outcome measurements such as the PRWE score or Disabilities of the Arm, Shoulder, and Hand score. None of the studies used a score derived specifically for the older population or evaluated changes in independence or living situation despite the fact that these are very relevant issues for older patients. Additionally, information on the quality of life after treatment is lacking. This study aims to compare the outcome of surgically and nonoperatively treated distal radius fractures in older patients by not only evaluating functional and radiological outcomes but also quality of life and change in independency or living situation. Furthermore, a functional score that was specifically designed to assess the physical activity level of older patients will be used.

One limitation of this study to consider is the relatively new study design. The ratio of opposing opinions among the expert panel to define equipoise was set at 2:4. One could argue that a ratio of 1:5 or 3:3 might be a better choice. A previous study performed with ethical committee members examined which proportion of agreement on the merit of a new treatment was perceived by the members as ethically justifiable to perform a trial investigating the new treatment. This study showed a distribution of 1:4 to suffice for the members to deem the trial ethically justifiable [20]. Based on the findings of that study in combination with an equal number of experts from both regions, the ratio of 2:4 was chosen for this study.

In conclusion, this observational study, designed as a natural study, will provide valuable information on treatment outcomes for those older patients with distal radius fractures for which to date equipoise exists regarding the optimal treatment. By evaluating outcomes pertinent to the older population, such as quality of life and changes in living situation or independence, this study will help tailor treatment to the specific needs of older patients.

## Abbreviations

PRWE: Patient-Rated Wrist Evaluation
REDCap: Research Electronic Data Capture
SPIRIT: Standard Protocol Items: Recommendations for Interventional Trial
